# Expanding the Known Repertoire of C-Type Lectin Receptors Binding to Toxoplasma gondii Oocysts Using a Modified High-Resolution Immunofluorescence Assay

**DOI:** 10.1128/mSphere.01341-20

**Published:** 2021-03-31

**Authors:** Benedikt T. Fabian, Bernd Lepenies, Gereon Schares, Jitender P. Dubey, Furio Spano, Frank Seeber

**Affiliations:** a Robert Koch-Institute, FG16: Mycotic and Parasitic Agents and Mycobacteria, Berlin, Germany; b Immunology Unit and Research Center for Emerging Infections and Zoonoses, University of Veterinary Medicine, Hannover, Germany; c Friedrich-Loeffler-Institut, Federal Research Institute for Animal Health, Institute of Epidemiology, National Reference Centre for Toxoplasmosis, Greifswald-Insel Riems, Germany; d United States Department of Agriculture, Agricultural Research Service, Beltsville Agricultural Research Center, Animal Parasitic Diseases Laboratory, Beltsville, Maryland, USA; e Department of Infectious Diseases, Istituto Superiore di Sanità, Rome, Italy; University at Buffalo

**Keywords:** *Toxoplasma gondii*, oocyst, C-type lectin receptor, immunofluorescence assay, dendritic cells, Apicomplexa

## Abstract

The environmental stage of the apicomplexan Toxoplasma gondii oocyst is vital to its life cycle but largely understudied. Because oocysts are excreted only by infected felids, their availability for research is limited. We report the adaptation of an agarose-based method to immobilize minute amounts of oocysts to perform immunofluorescence assays. Agarose embedding allows high-resolution confocal microscopy imaging of antibodies binding to the oocyst surface as well as unprecedented imaging of intracellular sporocyst structures with Maclura pomifera agglutinin after on-slide permeabilization of the immobilized oocysts. To identify new possible molecules binding to the oocyst surface, we used this method to screen a library of C-type lectin receptor (CLR)-human IgG constant region fusion proteins from the group of related CLRs called the Dectin-1 cluster against oocysts. In addition to CLEC7A that was previously reported to decorate T. gondii oocysts, we present experimental evidence for specific binding of three additional CLRs to the surface of this stage. We discuss how these CLRs, known to be expressed on neutrophils, dendritic cells, or macrophages, could be involved in the early immune response by the host, such as oocyst antigen uptake in the intestine. In conclusion, we present a modified immunofluorescence assay technique that allows material-saving immunofluorescence microscopy with T. gondii oocysts in a higher resolution than previously published, which allowed us to describe three additional CLRs binding specifically to the oocyst surface.

**IMPORTANCE** Knowledge of oocyst biology of Toxoplasma gondii is limited, not the least due to its limited availability. We describe a method that permits us to process minute amounts of oocysts for immunofluorescence microscopy without compromising their structural properties. This method allowed us to visualize internal structures of sporocysts by confocal microscopy in unprecedented quality. Moreover, the method can be used as a low- to medium-throughput method to screen for molecules interacting with oocysts, such as antibodies, or compounds causing structural damage to oocysts (i.e., disinfectants). Using this method, we screened a small library of C-type lectin receptors (CLRs) present on certain immune cells and found three CLRs able to decorate the oocyst wall of T. gondii and which were not known before to bind to oocysts. These tools will allow further study into oocyst wall composition and could also provoke experiments regarding immunological recognition of oocysts.

## INTRODUCTION

The apicomplexan intracellular parasite Toxoplasma gondii infects virtually all warm-blooded animals. However, sexual reproduction occurs only in the intestine of members of the Felidae family, resulting in the excretion of oocysts. These oocysts have been shown to be highly infectious (1) and can remain viable in the environment for months or years. Reliable methods able to provide good estimates of the numbers of oocysts present in the environment are scarce. Since cats usually shed oocysts only once, experimental access to oocysts is limited and only few laboratories worldwide have the capability to provide this important stage for the research community (2, 3). This has resulted in limited knowledge on its biology, including its biochemical makeup (4, 5).

Oocysts are ovoid and 11 to 13 μm in diameter (6). They contain two sporocysts, each harboring four sporozoites. The sporocysts are protected within the oocyst by a bilayered wall. The outer wall is approximately 31 nm thick and electron dense, whereas the inner wall is electron lucent and thicker at 61 nm (7). The inner wall is resistant to sodium hypochlorite degradation, while the thin outer layer is removed by this treatment (8). Both layers of the oocyst wall are likely interconnected by tyrosine-rich proteins, with a coat of acid-fast lipids on the outer surface of the oocysts. The outer wall is reinforced by a scaffold of cysteine-rich proteins and the inner wall by a scaffold of β-1,3 glucans (5, 9, 10). The presence of these β-1,3 glucans on the inner wall has been assumed based on binding of C-type lectin receptor (CLR) CLEC7A (alternative name, Dectin-1) (11). This CLR recognizes different β-1,3-linked and β-1,6-linked glucans in fungi as well as in plants (12).

CLEC7A is part of a cluster of CLRs called Dectin-1 cluster (12). This family of CLRs belongs to the so-called mammalian natural killer gene complex, is expressed in a broad range of immune cells, and detects diverse ligands, thus functioning in a wide variety of innate immune responses (13). Other CLRs forming this cluster are CLEC1A (CLEC-1), CLEC1B (CLEC-2), CLEC8A (LOX-1), CLEC9A (DNGR1), CLEC12A (MICL), and CLEC12B (*m*acrophage *a*ntigen *H* [MAH]) (12). CLEC7A and CLEC8A are involved in the detection of fungal and bacterial structures, respectively, and both play a role in the detection of apoptotic cells via their ligands. This detailed characterization does not apply to other members of the Dectin-1 cluster, for which there is only limited knowledge of the ligands of CLEC1B, CLEC9A, CLEC12A, and CLEC12B (see Table 1). This is partly due to the widespread promiscuous and flexible nature of binding sites of CLRs. It is best illustrated by two of the known ligands of CLEC1B, namely, the nonglycosylated snake venom toxin rhodocytin and the type I sialomucin-like glycoprotein podoplanin. Both lead to platelet aggregation upon binding to CLEC1B. However, binding is mainly mediated by amino acid side chains in the case of rhodocytin but in podoplanin by sialic acid interaction with charged amino acids of the binding pocket, as revealed by three-dimensional (3D) structural analyses (14). We reasoned that the identification of additional CLRs able to bind to the oocyst wall might reveal new insights not only into oocyst wall composition but also into the little studied early immune responses to infection with T. gondii oocysts (15, 16), thus prompting our investigations of more members of the Dectin-1 cluster.

**TABLE 1:** Investigated CLRs of the Dectin-1 cluster and their known ligands^*a*^

CLR	Known ligands (reference)
CLEC1B	Rhodocytin (84)
	Podoplanin (14)
	Fucoidan
	Diesel exhaust particles
CLEC7A	β-glucan
	Tropomyosin
	*Mycobacterium* (85)
	*Leishmania* (86)
	Galectin
	Galactosylated immunoglobulin
CLEC9A	Dead cells (F-actin, myosin II) (54)
CLEC12A	Dead cells (28)
	Hemozoin (61)
	Uric acid (28)
CLEC12B	Unknown

a Table was based on Tone et al. (12).

Although oocysts are precious for experimental purposes, there is a lack of established protocols describing the optimal use of minute amounts of this important stage. One such assay that is inherently suited for the study of single oocysts is the microscopic immunofluorescence assay (IFA). However, most current protocols for antibody incubations and washings use either air-dried oocysts fixed onto slides or tube-based oocyst suspensions (e.g., reference 17). While the former method leads to dehydration and possible collapse of internal structures resulting in artifacts, they can be prevented when incubations are performed in suspension. However, tube-based methods are characterized by a high loss of oocysts because they have a slight negatively charged surface (18) and tend to stick to the walls of centrifuge tubes. Thus, large amounts of starting material are required for a satisfying recovery of oocysts that can then be transferred onto slides for fluorescence microscopy. Moreover, the handling of live oocysts in order to preserve the integrity of the wall may increase the chances of accidental infection of the experimenter due to aerosol formation during tube opening and pipetting steps.

Here, we describe the adaptation and optimization of an IFA that entirely omits tube-based washing steps and uses only very small numbers of oocysts. This adaptation is based on the well-known concept of trapping or immobilizing living cells by an adhesive matrix like gelatin or agar/agarose directly on microscope slides (19, 20), which has been described previously to be compatible with fluorescence microscopy (21). Using such agarose-embedded oocysts (AEOs) allowed us to screen a CLR library, which was comprised of a variety of CLR-human IgG-Fc-fragment fusion proteins, covering several CLR clusters of human and murine origin (22, 23). We could thus identify additional CLRs from the Dectin-1 cluster binding to the outer oocyst surface. Moreover, this assay allowed us to visualize for the first time by fluorescence microscopy the sutures and their connecting points where the four plates of the sporocysts meet.

## RESULTS

### An agarose-based immobilization method allows IFAs with small numbers of oocysts.

We adapted and optimized a previously described IFA method for immobilization of peripheral blood mononuclear cells (21). Low-melt agarose (0.5%) provided a suitable matrix for embedding a small number of T. gondii oocysts on a microscopic slide. Furthermore, precoating slides with 1% low-melt agarose proved necessary and sufficient to prevent the embedded oocysts from washing off the slide. Small 3-μl drops were sufficient to achieve an even distribution of oocysts on the slides used (well diameter of 8 mm) and to allow agarose-embedded oocysts (AEOs) to be further examined on the slide with antibodies using fluorescence microscopy (termed AEO-IFA). Using fluorescent beads as a substitute for oocysts, we compared the numbers of beads before and after performing the IFA in a centrifuge tube versus embedding in 0.5% agarose on a microscopic slide. We observed only a minor loss of beads (<10%) using the agarose-embedded method, whereas incubation in a centrifuge tube and washing steps by centrifugation and pipetting resulted in a loss of more than 55% of beads (Table 2). At the same time, the number of beads needed in order to generate satisfying results as well as the overall assay volume was much lower in the slide-based method than that in the tube-based method. Thus, the AEO-IFA format provides a simple, miniaturized, and resource-saving alternative to current methods.

**TABLE 2:** Comparison of tube-based and agarose-based IFA methods

Method	Before incubation (total no. of beads)	After 2-h incubation and two washing steps (% of beads lost ± SD [*n*])	Assay vol (μl)
Centrifuge tube	6,750	56.8 ± 6.3 (3)	100
Agarose-embedded IFA	100	9.7 ± 8.3 (6)	10

### AEO-IFA allows identification of antibodies binding to the oocyst wall.

To confirm the suitability of our IFA method to detect antibodies binding to the oocyst wall, we incubated AEO with the 3G4 murine IgM monoclonal antibody (MAb) that was shown previously to specifically bind to the outer surface of oocysts and sporocysts but not sporozoites (24). Note that in this and all subsequent experiments, oocysts were used that had not been treated with 10% sodium hypochlorite since exposure to bleach is known to result in the removal of the outer wall layer (8).

**FIG 1:**
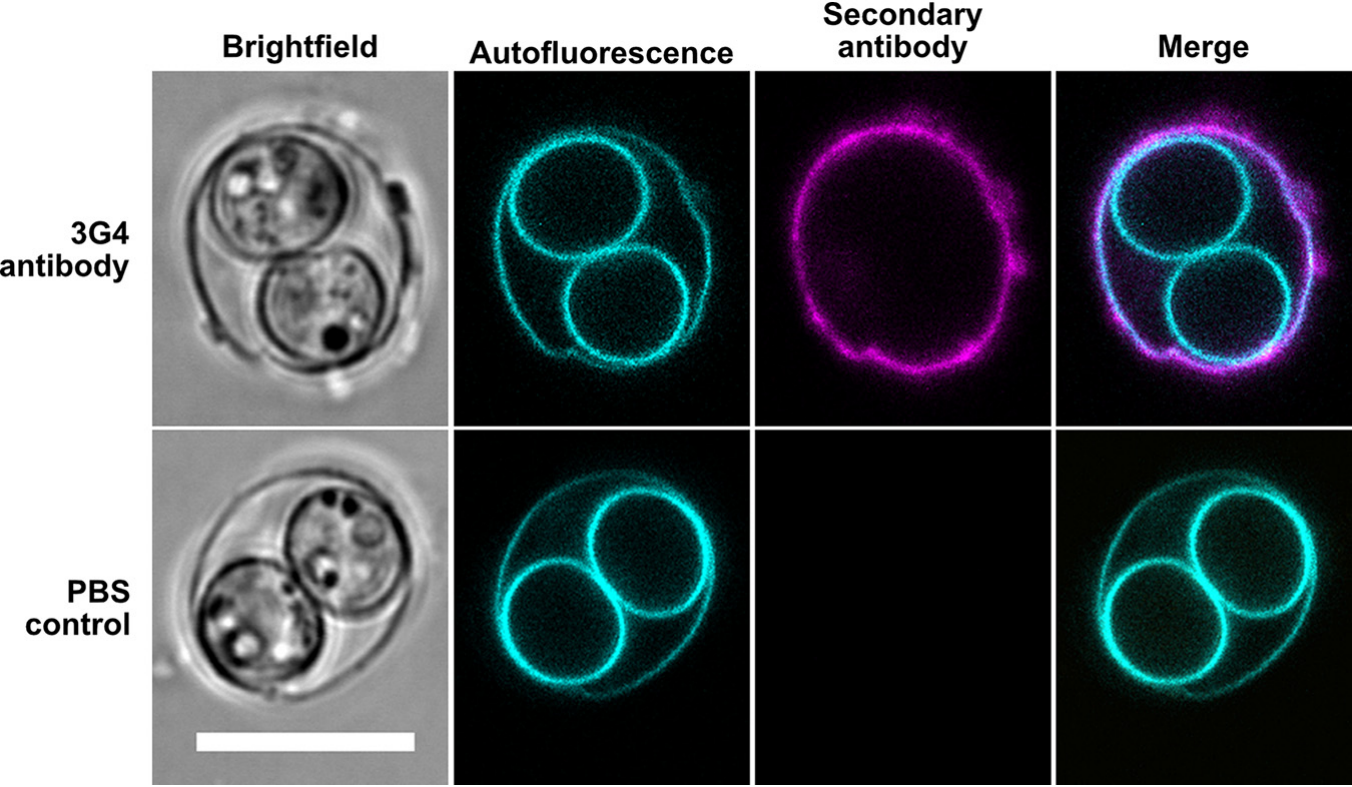
Immunofluorescence microscopy confirms successful binding of 3G4 antibody to the oocyst wall. Oocyst wall and sporocyst wall are visible by their autofluorescence, while detection of 3G4 antibody with Cy5-labeled secondary antibody reveals only the oocyst wall. Single planes of the same *z* axis are shown. For color-blind-friendly visualization, colors of the fluorescent channels were adjusted as follows: blue to cyan and red to magenta. Scale bar = 10 μm.

As shown in Fig. 1, autofluorescence of T. gondii oocysts (due to tyrosine-rich proteins [5]) marks the oocyst wall as well as the outline of the sporocysts. Immunofluorescence of intact oocysts confirmed the specific binding of MAb 3G4 to its outer surface (Fig. 1, top), indicating unlimited access of molecules through the agarose. No unspecific background signals due to insufficient removal of unbound large IgM antibodies could be observed.

### AEO-IFA allows analysis of molecules binding to inner oocyst structures.

**FIG 2:**
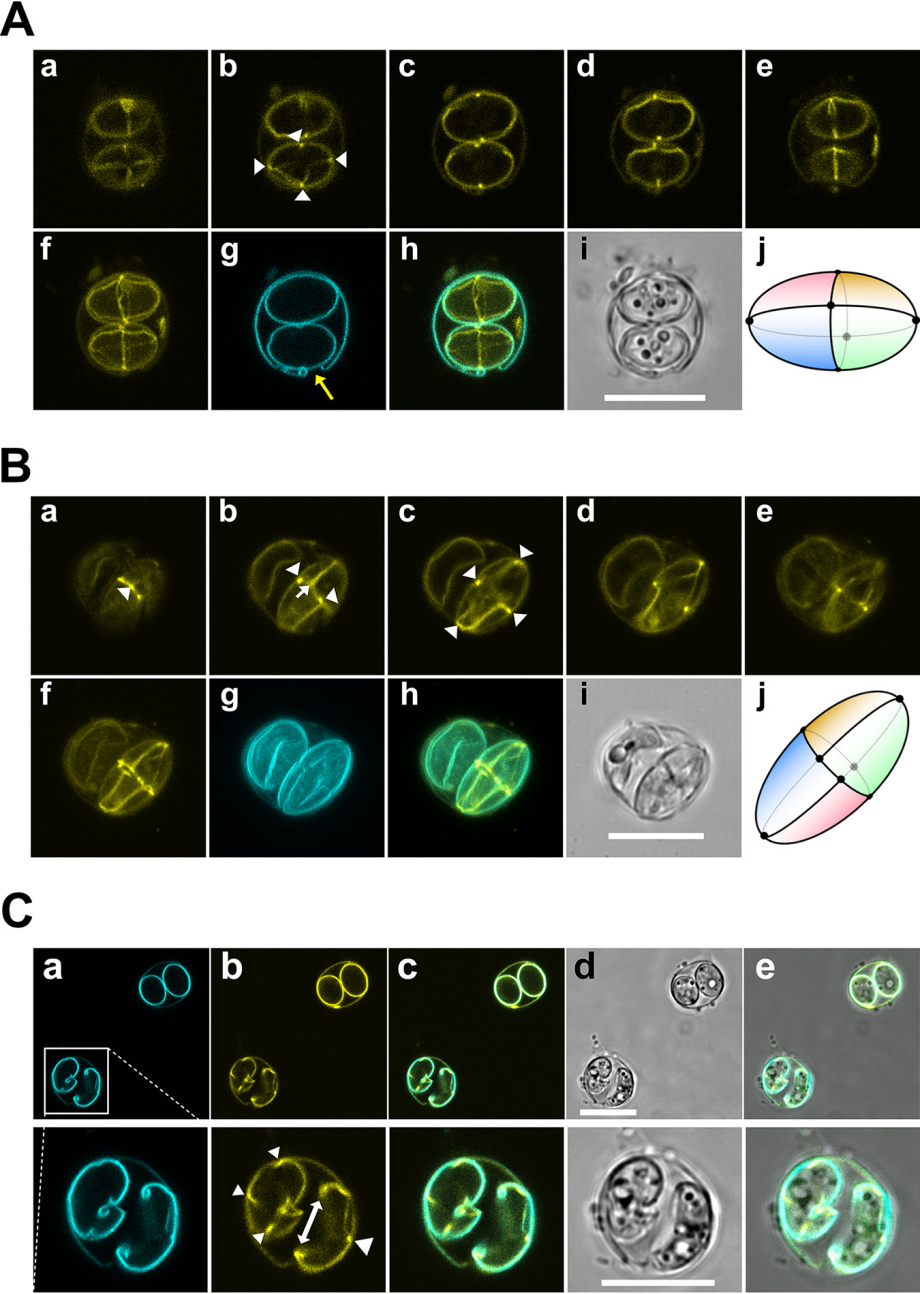
Immunofluorescence microscopy images of oocyst inner structures. Shown is binding of MPA-FITC to sporocyst structures without (A) or with prior acetone treatment (B, C). For color-blind-friendly visualization, colors of the fluorescent channels were adjusted as follows: blue to cyan and green to yellow. Note in all images the increased staining by MPA of the sutures where the four plates making up the sporocyst shell meet (arrowheads). (A, B) The yellow arrow in Ag points to an opening of the oocyst wall. Fluorescence images a to e are single planes of a z-stack; in f are *z* axis projections of the stack; in g in A it is a single plane of the autofluorescence z-stack, whereas in B it is a z-projection of the stack; h shows merged image of f and g; i shows focus-stacked brightfield image; j shows scheme of the four plates and their orientation with regard to the midline suture, as deduced from the MPA-stained sutures (a to f) and their corresponding connecting points. (C) MPA staining of acetone-treated oocysts allows refined observation of sporocyst opening due to the increased staining at the sutures. Dashed lines indicate zoomed-in representations (bottom row) of oocysts in the boxed area (top row). Scale bars = 10 μm.

Given the fact that the oocysts available to us had been inactivated either by heat treatment or freeze-thaw cycles (see Materials and Methods) and were thus assumed to be at least partially permeable to larger molecules, we assessed whether the AEO-IFA also allowed the visualization of internal structures. Embedded oocysts were therefore incubated on the slide with fluorescein isothiocyanate (FITC)-conjugated Maclura pomifera agglutinin (MPA) that is known to bind to glycoproteins present in the oocyst as well as the sporocyst wall of T. gondii (5, 9) (Fig. 2A and B). Fluorescent MPA outlined the sporocysts inside the oocyst and clearly emphasized the sutures and their connecting points where the four plates making up the sporocysts meet (Fig. 2A, b, arrowheads, j) (7, 25). To our knowledge, these structures have not been detected in previous observations of MPA-labeled oocysts (9), which underscores an advantage of AEO-IFA. In an attempt to increase the walls’ permeabilization, we treated embedded oocysts on the slide with ice-cold acetone before MPA incubation (Fig. 2B). A somewhat enhanced staining of sutures was observed since only one-half of the laser intensity was required to obtain a comparable signal to that shown in Fig. 2A, which is indicative of increased permeabilization. Compared with autofluorescence (Fig. 2Ca), staining with MPA also allowed refined imaging of sporocyst openings, reminiscent of excystation (Fig. 2Cb). However, since we did not follow a strict excystation protocol, we cannot rule out that the observed sporocyst opening process could have been also caused by H_2_SO_4_ or acetone treatment or the freeze-thaw cycles used for inactivation rather than due to excystation events. The left sporocyst is in the early stage of opening, where the junctions of the plates are just beginning to curl inward. In the right sporocyst, this process already resulted in a large opening in the wall on one side (double arrow, Fig. 2Cb, bottom). The connecting point of the other plates at the opposing side and where opening and rolling-in supposedly starts is clearly demarcated by intense MPA staining (right arrowhead, Fig. 2Cb, bottom). These results mirror similar earlier findings at the ultrastructural level describing sporozoite excystation (7, 26).

**FIG 3:**
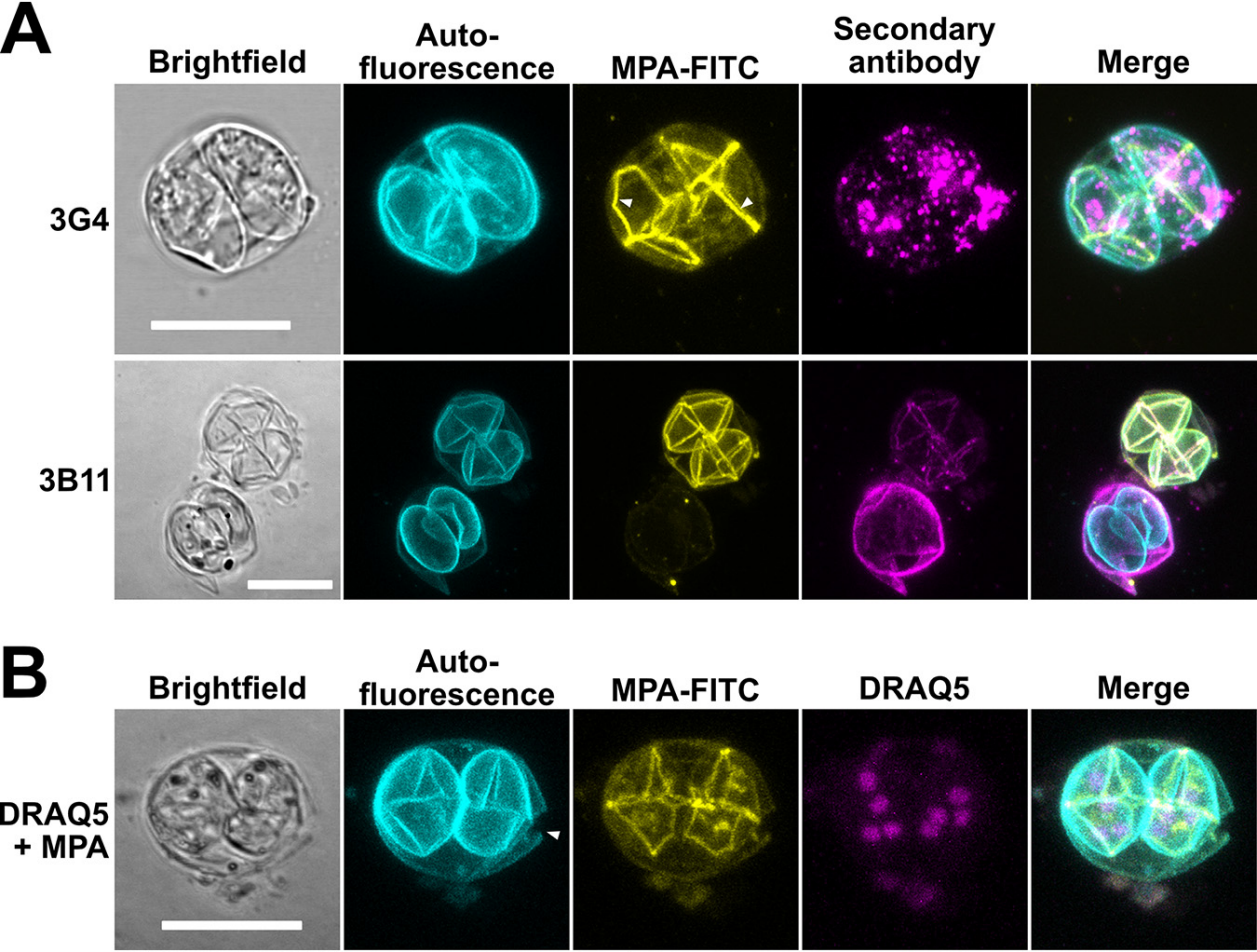
Fluorescence microscopy reveals different effects of acetone treatment on antibody binding (A) and successful staining of nuclei within each of the two sporocysts (B). (A) Acetone treatment of oocysts resulted in loss of defined signal for MAb 3G4 compared with Fig. 1, whereas TgOWP3-specific MAb 3B11 binding is less affected. (B) DRAQ5 visualization of four nuclei per sporocyst. In the absence of DRAQ5, no stained nuclei were observed (data not shown). MPA-decorated sporocyst sutures indicate permeabilization of oocysts. In addition, the oocyst seemed to be ruptured (arrowhead). Fluorescence images are *z* axis projections of stacks; brightfield images represent either a singular plane from the same stack or a focus-stacked image. For color-blind-friendly visualization, colors of the fluorescent channels were adjusted, as follows: blue to cyan, red to magenta, and green to yellow. Scale bars = 10 μm.

The effect of acetone treatment was not always uniform and predictable, however. This is illustrated by MAb 3G4 reactivity. It resulted in the loss of the distinct surface staining and instead in an intense, uneven and spotty signal within the sporocysts (Fig. 3A, top; compare with Fig. 1). To see whether this was a general phenomenon of acetone treatment, we used another MAb, 3B11 (for its generation and characterization, see Materials and Methods; see Fig. S1 in the supplemental material), obtained from mice immunized with recombinant outer wall protein 3 of T. gondii (TgOWP3) and known to be part of the oocyst wall (10). While acetone treatment did not influence the ability of MAb 3B11 to bind to many oocysts, not all walls were consistently stained, and weak reactivity of 3B11 with the sporocyst sutures could be observed in those cells where MPA staining indicated permeabilization (Fig. 3A, bottom). Furthermore, when we assessed nuclei staining within the oocyst upon acetone permeabilization (Fig. 3B), again some inconsistencies were frequently observed within the same slide, irrespective of the stain used (see Fig. S2 in the supplemental material). The reasons for this heterogeneity within our oocyst population are currently unknown. Besides differences in the oocyst age, one explanation could be the dehydrating effect of acetone, leading to the collapse of the oocyst/sporocyst structure, as has been observed previously by EM (27). It might cause aberrant signals due to a partial loss of structural integrity, which could also explain the limited spatial resolution of air-dried specimens in previous studies. Taken together, these results show that acetone treatment is possible and allows high-resolution confocal fluorescence microscopy of internal structures of permeabilized oocysts. It neither affected permanent entrapment of oocysts in the agarose matrix nor led to a noticeable decrease in their autofluorescence. However, its effect on antigen preservation and/or effectiveness on permeabilization requires individual assessment and proper controls.

10.1128/mSphere.01341-20.2 FIG S1 Reactivity of anti-TgOWP3 monoclonal and polyclonal antibodies with T. gondii oocysts by IFA. T. gondii oocysts of the type III strain VEG, treated with 10% sodium hypochlorite prior to staining, were air-dried on glass slides, fixed with cold ethanol, and incubated with either the anti-TgOWP3 MAbs 3B11 or with an anti-TgOWP3 mouse polyclonal serum (10). The hybridoma supernatant containing a MAb directed against an unrelated histidine-tagged protein antigen was used as a negative control. Oocyst-bound primary antibodies were revealed by an Alexa 594-conjugated goat anti-mouse secondary antibody. For color-blind-friendly visualization, colors of the fluorescent channels were adjusted, as follows: blue to cyan and red to magenta. Scale bar = 10 μm. Download FIG S1, TIF file, 0.8 MB. Copyright © 2021 Fabian et al. 2021 Fabian et al. https://creativecommons.org/licenses/by/4.0/ This content is distributed under the terms of the Creative Commons Attribution 4.0 International license.

10.1128/mSphere.01341-20.3 FIG S2 Fluorescence microscopy images of oocysts with 4′,6-diamidino-2-phenylindole (DAPI) in the mounting medium show only partial staining of nuclei. In some cases where nuclei were visible, this was only the case for one of the two sporocysts per oocyst, whereas some other oocysts were not stained at all. The image is a *z* axis projection of a stack of 30 images. For consistency with all other images the color of the blue fluorescent channel was changed to cyan. Scale bar = 10 μm. Download FIG S2, TIF file, 0.2 MB. Copyright © 2021 Fabian et al. 2021 Fabian et al. https://creativecommons.org/licenses/by/4.0/ This content is distributed under the terms of the Creative Commons Attribution 4.0 International license.

### Screen of a CLR library identifies new oocyst-binding Dectin-1 members.

**FIG 4:**
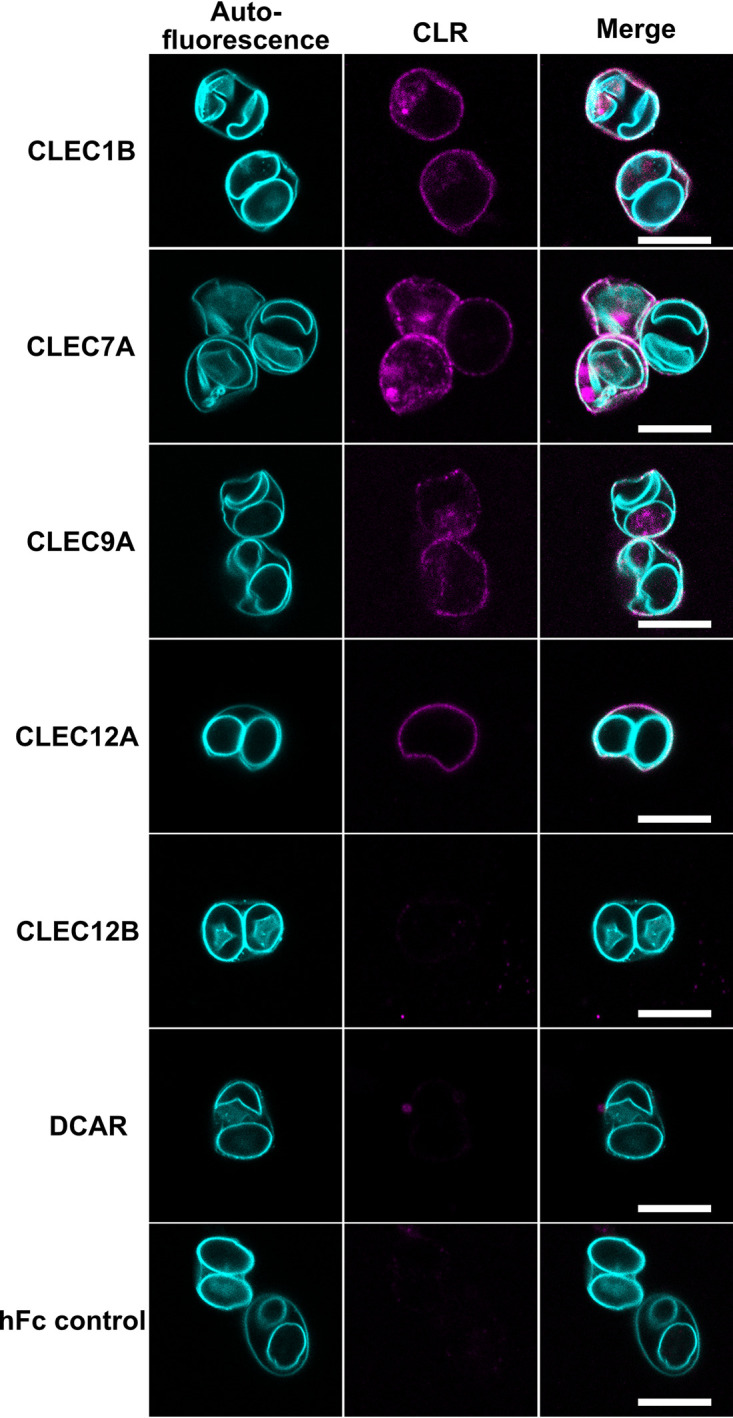
Screen of CLRs of the Dectin-1 cluster reveals CLRs binding to oocysts. The left column shows autofluorescence of AEO, the center column shows a signal of DyLight-650-labeled anti-human IgG antibody in case of CLR binding, and the right column is a merge of the two images. No binding was observed for CLEC12B, DCAR, and the hFC control. See also Fig. S3. All microscopic images were obtained with identical settings for laser intensities. Brightness and contrast in merged images were adjusted equally for better visibility. For color-blind-friendly visualization, colors of the fluorescent channels were adjusted, as follows: blue to cyan and red to magenta. Scale bar = 10 μm.

Next, we applied the AEO-IFA to search within the Dectin-1 cluster for potential CLRs binding to T. gondii oocysts, employing a murine CLR-hFc fusion protein library. The chimeric CLR-hFc fusion proteins consist of the extracellular part of the respective murine CLR covering the carbohydrate recognition domain, fused to the Fc fragment of human IgG_1_ molecules (22). The following Dectin-1 CLRs of murine origin present in the library were analyzed: CLEC1B, CLEC7A, CLEC9A, CLEC12A, and CLEC12B. In addition, we also investigated a CLR not from the Dectin-1 cluster, DCAR. As a negative control, the hFc fragment not containing any CLR domain was used in all assays. AEO-IFA confirmed CLEC7A binding, which was used as a positive control (11), and identified CLEC1B, CLEC9A, and CLEC12A as newly recognized CLRs capable of decorating the oocyst wall (Fig. 4; Fig. S3).

### CLRs bind specifically to the oocyst wall of T. gondii and do not discriminate between sporulated and unsporulated oocysts.

**FIG 5:**
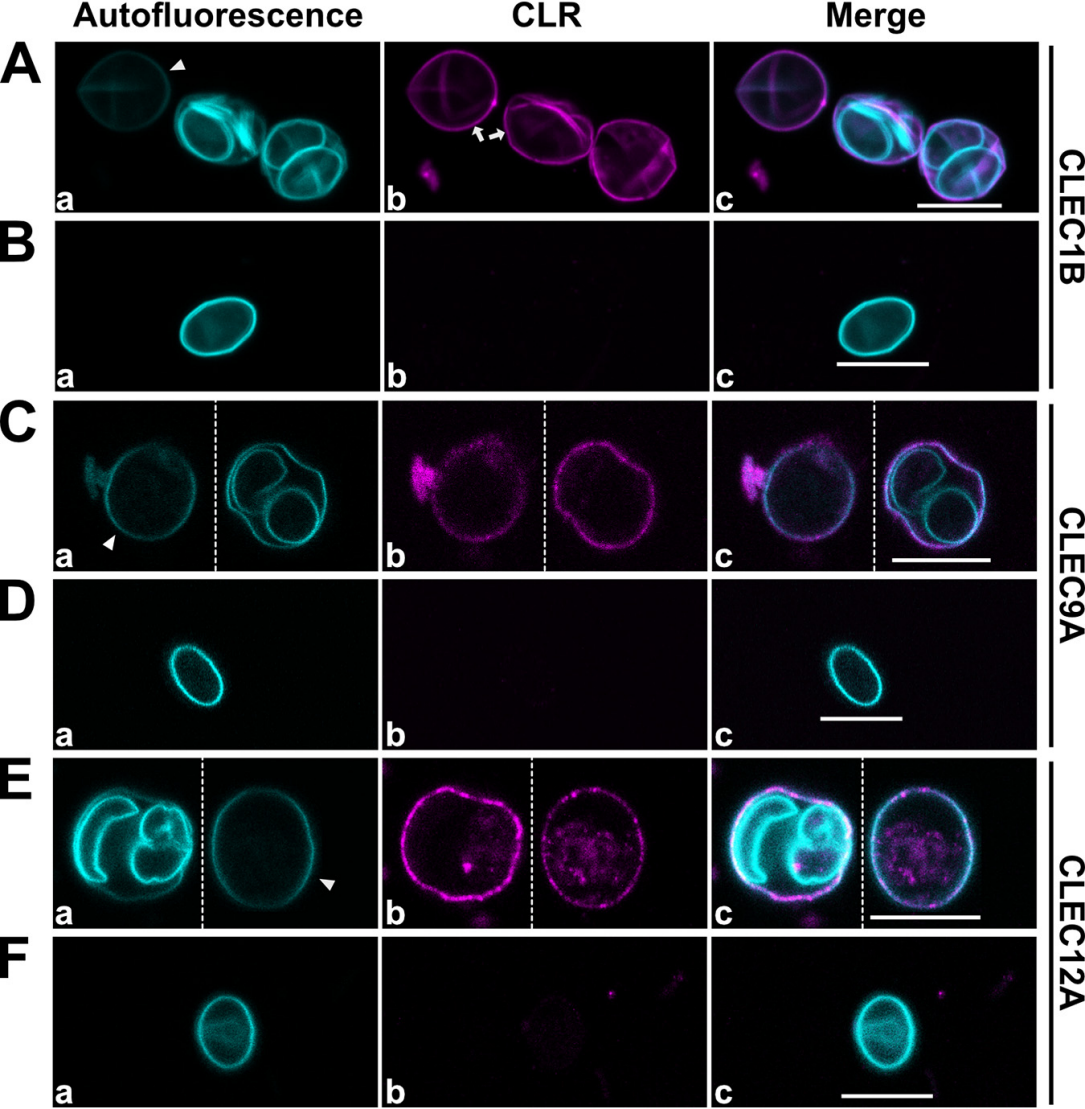
Immunofluorescence microscopy images show equivalently good binding of CLRs to the oocyst wall of sporulated and unsporulated oocysts. (A, C, E) The CLRs binds to both sporulated and unsporulated oocysts. Note the decreased autofluorescence of the unsporulated oocysts (arrowheads, a), while the DyLight-650 signal of the secondary antibody does not differ between the oocysts (arrows, b). (B, D, F) Sporocyst walls are not detected by the CLRs, as confirmed by absence of DyLight-650 signal in the merged images (c). For better visibility, oocysts from the same field of view in the original image have been cropped and placed next to each other to allow image enlargement (indicated by stippled lines). Also, brightness of b and c in E has been slightly increased. For color-blind-friendly visualization, colors of the fluorescent channels were adjusted, as follows: blue to cyan and red to magenta. All scale bars = 10 μm.

We analyzed the newly identified CLR binding to oocysts more in depth. None of the CLRs bound to cysts of Giardia duodenalis or *Cryptosporidium* sp. oocysts under similar conditions (data not shown). However, CLEC1B evenly decorated sporulated and unsporulated T. gondii oocysts (Fig. 5A). Furthermore, we could not detect binding of the CLR to the sporocyst wall when ruptured oocysts were observed by IFA (Fig. 5B). The same observations were also made for CLEC9A and CLEC12A (Fig. 5C to F).

### CLEC12A ligand on T. gondii oocysts is not uric acid.

**FIG 6:**
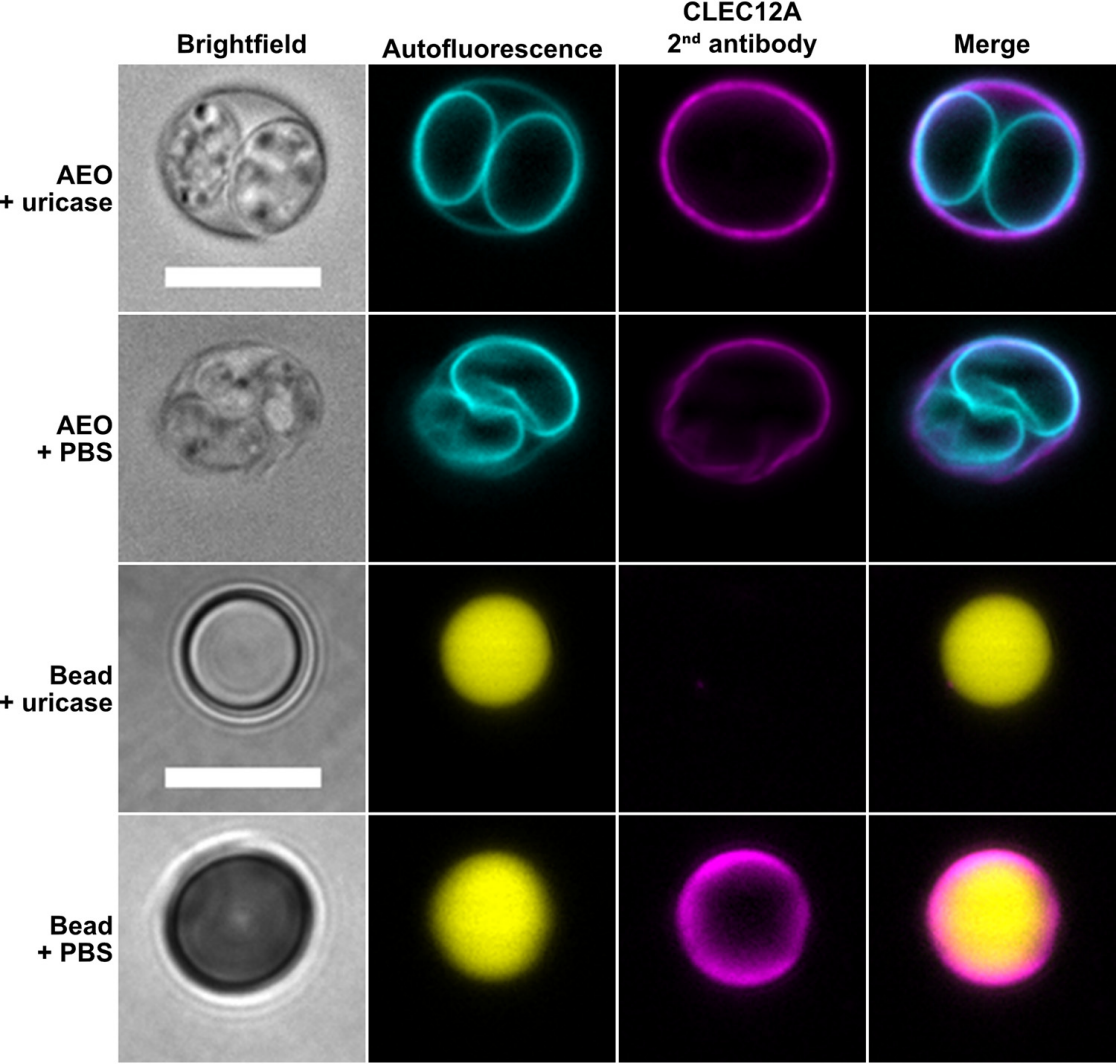
Incubation with uricase prior to CLEC12A rules out binding of CLEC12A to uric acid crystals on oocysts. The top two panels depict binding of CLEC12A to AEO with or without uricase treatment prior to the IFA. DyLight-650 signal of the secondary antibody is not reduced upon uricase treatment in the second to right picture. The bottom two panels depict fluorescent beads coated with uric acid crystals and treated or not treated with uricase prior to the IFA, respectively, resulting in the absence of DyLight-650 signal upon uricase treatment. For color-blind-friendly visualization, colors of the fluorescent channels were adjusted, as follows: blue to cyan, red to magenta, and green to yellow. Scale bars = 10 μm.

One known ligand of CLEC12A is uric acid crystals (28). Uric acid is produced by cellular xanthine oxidase and released after cell injury. Upon contact with Na^+^, it forms monosodium urate microcrystals, thereby acting as an alarmin for cell death (29, 30). To rule out the possible contamination of cat feces and thereby T. gondii oocysts with urine-derived uric acid, we pretreated oocysts with uricase prior to CLEC12A incubation. As shown in Fig. 6, this pretreatment did not inhibit binding of CLEC12A to the outer oocyst surface. To confirm successful uric acid degradation by uricase, fluorescent beads were added to uric acid crystals overnight and subsequently treated with uricase prior to incubation with CLEC12A. This procedure resulted in a loss of signal compared with untreated beads, where CLEC12A decorated the uric acid crystals present on the bead surface. We conclude that uric acid crystals are not responsible for CLEC12A binding to T. gondii oocysts, suggesting the presence of a specific, yet unknown ligand on the oocyst wall.

## DISCUSSION

Recently, linoleic acid was identified as the factor limiting sexual reproduction in felid hosts, allowing the generation of T. gondii oocysts in intestinal organoids or mice (31). However, only a few oocysts were formed in mice, and they were fragile. Therefore, there is a dire need for methods reducing the number of these stages required for experimentation or analyses.

Our rationale for establishing the AEO-IFA built upon prior experience with cell immobilization using low-melting agarose (19, 21, 32). The AEO-IFA has a number of advantages over other hydrogels that have been described for this purpose, like alginate (33, 34) or acrylate copolymers (35). The latter require handling of poisonous chemicals and sophisticated balancing of the ingredients for best results. Alginate hydrogels need optimization regarding the choice of divalent ion and its concentration or the ratio of ions-to-alginate for optimal gelling. In contrast, low-melting agarose is simply dissolved and heated in any buffer of choice, and the gelling strength is adjusted by agarose concentration. One of the biggest advantages of agarose is its chemical and physical inertness (36), for instance to acetone. Acetone treatment does not lead to significant water loss of agarose gel over time (37), whereas alginate gels shrink by 75% and also change color (38). Consequently, antibody access to the inner part of the agarose gel and solute exchange are not impaired, as would probably be the case for alginate-embedded oocysts. However, water removal by acetone might apply some pressure onto the embedded oocysts (dimension, 11 by 13 μm) within the pores of the gel (0.6 to 1 μm) (39), leading to collapse under dehydration conditions (27). Still, in the case of our AEO-IFA, we considered acetone as a permeabilization agent that was advantageous over fixatives, such as methanol or Triton X-100, due to reports of poor retention of antigenicity (40) or no apparent effect on oocyst survival and thus permeabilization (41), respectively. Nevertheless, acetone should be used with care and requires proper controls, such as a comparison with no or alternative fixation methods. It is known to have profound effects on the detection of cellular structures, in particular membrane-associated antigens (42).

Overall, the AEO-IFA method we present here requires only minute amounts of T. gondii oocysts and will be useful for the phenotypic analysis of the limited numbers of oocysts obtainable from aforementioned alternatives to cat infections. It allows investigations of the ligands of lectins, antibodies, and other binding molecules, without compromising high-resolution confocal fluorescence microscopy. This is exemplified by the visualization, for the first time, by light microscopy of the sutures of the four plates that make up the inner layer of the sporocyst (Fig. 2). Consistent with the few available scanning electron microscopic images of this structure (5, 7, 25), the orientation of the plates can be either along a straight longitudinal line (Fig. 2Aj) or somewhat shifted along the midline (Fig. 2Bj). Its relevance, if any, is unknown.

Preincubation with 100 mM of the MPA ligand *N*-acetyl-galactosamine (or *N*-acetylglucosamine as control) did not lead to an observable significant reduction of stained sporocyst sutures nor sporocyst walls (data not shown). While this lack of binding inhibition might seem surprising, given reports with other protozoans describing successful competition of MPA binding to surface-exposed ligands with *N*-acetyl-galactosamine (43–45), others saw little effect (46). Carbohydrate-mimetic peptides binding to lectins are known (47), and peptides mimicking the strong-binding MPA ligand Gal(β1-3)GalNAc(α1-*O*-Ser/Thr), also called TF-antigen (48), have been described (49). The three-dimensional structure of tetrameric MPA indicates the potential for lattice formation, which implied the possibility for more complex interactions with other molecules like amino acids (48, 50), expanding the repertoire of potential ligands beyond glycans. Thus, MPA binding to the sporocyst wall could also be due to interactions with noncarbohydrate ligands, but this will require further studies.

By applying our AEO-IFA to the screening of a small library of different CLRs of murine origin, we identified three new CLRs binding to the oocyst wall, namely, CLEC1B, CLEC9A, and CLEC12A, which all belong to the Dectin-1 cluster (Table 1).

CLEC1B, like CLEC7A, carries a similar immunoreceptor tyrosine-based activation motif in its cytosolic domain, proposing a similar physiological function upon binding of a ligand, such as the snake venom polypeptide rhodocytin, the *O*-glycosylated glycoprotein podoplanin, or the sulfated polysaccharide fucoidan. While the first analyses based on transcriptomic data indicated that CLEC1B is primarily present on the surface of platelets, newer findings suggest a more widespread expression. Kerrigan et al. demonstrated that CLEC1B is also expressed by neutrophils where it induces phagocytosis upon binding of rhodocytin (51). Furthermore, the authors could show that CLEC1B activation induced expression of tumor necrosis factor alpha (TNF-α) in these neutrophils. However, a candidate parasite protein ligand on oocysts similar to podoplanin or rhodocytin is so far unknown and not apparent in the genome-derived proteome in ToxoDB. Likewise, we could not find by BLAST searches any evidence for a sulfotransferase gene (52), the terminal enzyme required for the synthesis of sulfated glycans like fucoidan, making it unlikely that it is the ligand for CLEC1B.

CLEC9A, among others, is expressed on BDCA3^+^ dendritic cells and monocyte subsets. In infections by another apicomplexan, Plasmodium falciparum, an absence of CLEC9A^+^ dendritic cells led to a strong reduction in brain CD8^+^ T cells, which was accompanied by resistance to experimental cerebral malaria (53). It is an activating CLR, and its only known ligand is present on dead cells, where it binds to exposed F-actin (54, 55), a main component of the cytoskeleton, which is released upon cellular necrosis. Acting as a receptor for necrotic cells (56, 57), the main role of CLEC9A is the cross-presentation of antigen (58). This can be further amplified by the activity of myosin II (59). Interestingly, in the T. gondii-related coccidian Eimeria maxima F-actin was shown to be located underneath the outer oocyst wall (60). However, in preliminary experiments, we could not observe binding of phalloidin to T. gondii oocysts, indicating that CLEC9A binding in our assays is not due to F-actin being accessible to this protein (data not shown).

Another CLR involved in *Plasmodium* infections is CLEC12A, where it binds to hemozoin, a heme degradation product specific to this apicomplexan, resulting in CD8^+^ T cell-mediated cross-priming (61). The molecule(s) that CLEC12A binds to on T. gondii oocysts remain(s) unknown. However, we could rule out uric acid crystals, another known ligand of CLEC12A (28), as an interaction partner.

CLEC7A and CLEC12A are both expressed on the surface of several cell types, and among them are myeloid dendritic cells and macrophages. CLEC7A engagement stimulates immune cell activation through binding to particulate rather than soluble β-glucans (62). It results in induction of the cellular phagocytosis machinery, generation of reactive oxygen species, and inflammasome activation (63). Previous *in vitro* studies showed that oocysts are taken up by a mouse macrophage cell line in a phagocytic manner, and evidence was provided for subsequent oocyst wall rupture and occasional sporozoite release and intracellular differentiation into tachyzoites (64, 65). Freppel et al. speculated that oocyst phagocytosis might be a way of parasite dissemination in the host if similar processes with inhaled oocysts would occur in alveolar macrophages. However, solid evidence for airborne oocyst transmission has been hard to obtain (66, 67), although such laboratory-acquired infections were reported (68). CLEC7A and CLEC12A could be shown to be expressed on the surface of alveolar macrophages (69, 70). Hence, it would be interesting to see whether both CLRs are involved in oocyst uptake in this cellular system.

However, the much more relevant entry route of oocysts into a host is via the intestine, from where sporozoite-derived tachyzoite stages disseminate throughout the body (15). Strikingly, while antibody responses against oocyst proteins have been described (71–73), an immunological explanation for how components of the short-lived oocyst stage in the intestinal lumen could be presented to immune effector cells remains elusive. In this regard, an interesting observation is the coexpression of CLEC12A and the fractalkine receptor CX3CR1 on several dendritic cells and macrophages/monocytes (74–77). Intestinal macrophages expressing CXC3CR1 are known to sample antigens by extruding transepithelial dendrites (TEDs) into the intestinal lumen, without actually harming the integrity of the epithelium (78–80). Inhibition of TED extrusion was shown to lead to a lack of antibody responses and immunity against bacterial infection in mice (81). In the light of our results, we propose a testable hypothesis that postulates the uptake of remnants of oocyst walls in a CLEC12A-dependent manner by macrophages positive for the fractalkine receptor CX3CR1.

In conclusion, using our material-saving AEO-IFA allowing high-resolution imaging as part of low- to medium-throughput screening assays, we have greatly expanded the set of known Dectin-1 cluster CLRs binding to T. gondii oocysts. While the ligands for CLEC1B, CLEC9A, and CLEC12A on this parasite stage are currently unknown, the recombinant lectin receptor proteins will be useful tools in biochemical analyses for identifying these ligands. This would help to understand the composition of the oocyst wall and its resilience to harsh environmental conditions. Moreover, the implications of different CLRs in phagocytosis, antigen cross-presentation, and immune cell activation provoke interesting new hypotheses concerning early immune responses against the short-lived oocyst stage.

## MATERIALS AND METHODS

### Materials.

Yellow fluorescent beads used as surrogate microspheres were bought from Spherotech, USA (TFP-7052-5). The 3G4 MAb binding to the oocyst surface and FITC-labeled MPA (Vector Laboratories FL-1341) were a kind gift of Aurélien Dumètre, University of Marseille. The Cy5-labeled goat anti-mouse IgM antibody (115-175-075) was obtained from Dianova (Hamburg, Germany). The CLR-hFc fusion protein library we used has been published previously (23, 82). Briefly, cDNA fragments encoding the extracellular domain of the respective CLR were amplified by PCR using specific primers and ligated into a pFuse-hIgG_1_-Fc expression vector (InvivoGen, France). The CLR-hFc fusion proteins were expressed in CHO-S cells and purified from the supernatant using a HiTrap protein G column (GE Healthcare, USA). CLR-hFc purity and identity were confirmed by SDS-PAGE and subsequent Coomassie staining and Western blot by a horseradish peroxidase-conjugated anti-human IgG antibody (Dianova). The antibody for detection of CLR fusion proteins was a DyLight-650 labeled goat anti-human IgG Fc antibody (GtxHu-004-E650NHSX) from Dianova. Crystalline uric acid (U2625-25G) and uricase from *Candida* sp. (U0880-250UN) as well as Fluoromount^T^ (F4680-25ML) were from Sigma-Aldrich, USA. Mowiol was prepared by dissolving 9.6 g Mowiol 4-88 (81381; Sigma-Aldrich) in 24 g glycerol (7338.2; Carl Roth, Germany), 48-ml 0.2 M Tris buffer, and 24 ml distilled water. The six-well glass microscopic slides (1215130) and 12-mm coverslips (0111520) used for the AEO-IFA were obtained from Paul Marienfeld KG, Germany. For the tube-based IFA, we used low-bind reaction tubes (3207) from Corning Costar, USA. Fluorescent beads were counted in a Neubauer improved counting chamber. All other chemicals used (acetone [9372.1], bovine serum albumin [BSA; 8076.5], CaCl_2_, HEPES, MgCl_2_ and low-melt agarose [6351.5]) were from Carl Roth.

### Generation and characterization of monoclonal antibody 3B11.

MAb 1G12 (IgM) and a mouse polyclonal serum raised against a six-histidine-tagged recombinant version of TgOWP3 (recOWP3) were described previously (10), and the same protocols were used to obtain an additional TgOWP3-specific IgG isotype MAb, 3B11, described here. To this end, undiluted hybridoma supernatants were assayed in duplicate by ELISA on immobilized recOWP3. The polyclonal anti-OWP3 serum (1:2,000 dilution) was used as a positive control. The negative control was represented by the undiluted supernatant of a hybridoma, producing a MAb against an unrelated histidine-tagged protein (see Table S1 in the supplemental material). Isotyping of MAb 3B11 was done with a monoclonal antibody isotyping kit (Pierce). The surface reactivity of the antibodies was tested by IFA on T. gondii oocysts of the type III VEG strain and treated with 10% sodium hypochlorite prior to staining. Oocysts were air-dried on glass slides, fixed with cold ethanol, and incubated with either the anti-TgOWP3 MAb 3B11 (10) or with an anti-TgOWP3 mouse polyclonal serum (1:500 dilution) (Fig. S1). The hybridoma supernatant containing a MAb directed against an unrelated histidine-tagged protein antigen was used as negative control. Oocyst-bound primary antibodies were revealed by an Alexa 594-conjugated goat anti-mouse secondary antibody (1:1,000 dilution; Invitrogen, USA).

10.1128/mSphere.01341-20.1 TABLE S1 ELISA and immunofluorescence reactivity of anti-TgOWP3 antibodies. Download Table S1, DOCX file, 0.02 MB. Copyright © 2021 Fabian et al. 2021 Fabian et al. https://creativecommons.org/licenses/by/4.0/ This content is distributed under the terms of the Creative Commons Attribution 4.0 International license.

### Oocysts.

We used oocysts of T. gondii strain ME49 for all AEO-IFA experiments and which had not been treated with bleach. They had been generated by one of the authors (J. P. Dubey) in February 2018 at USDA, Beltsville, Maryland, and shipped via courier to G.S. at Friedrich-Loeffler-Institut, Greifswald-Insel Riems, Germany. Oocysts were divided into two batches, which were both inactivated prior to use. One batch was incubated at 60°C for 1 minute, while the other was frozen and then thawed at room temperature (RT) three times using an ethanol ice bath. Both batches were then stored separately at 4°C in 2% H_2_SO_4_ until use. Before experimentation, aliquots of both batches were pooled, washed three times using phosphate-buffered saline (PBS), and counted (∼250 oocysts per well were used).

### Tube-based IFA.

Similar amounts of fluorescent polystyrene beads (mean diameter of 7.0 to 7.9 μm) were distributed in 10-μl aliquots over 3 individual low-bind reaction tubes (6,750 beads per tube). A total of 90 μl of PBS containing 1% BSA (PBS-B) was added, and the tubes were incubated for 1 h at room temperature and 300 rpm. After incubation, the beads were centrifuged at 4,000 rpm for 5 minutes and the supernatant discarded. A total of 100 μl PBS was added, and this washing step was repeated two more times. Then, the beads were again incubated as described above, followed by the same washing steps. In a final step, the volume in the tubes was adjusted to 100 μl in PBS. For the final analyses of bead loss, 10-μl aliquots were counted in a Neubauer improved counting chamber.

### AEO-IFA procedure.

Individual wells of six-well glass microscopic slides were coated with 10 μl of 1% (wt/vol) low-melt agarose dissolved in Millipore-grade water and air-dried, after which they were stored at 4°C until use. Oocysts in PBS (2.5 × 10^5^/ml) and 1% low-melt agarose were mixed 1:1 (vol/vol) prior to being loaded onto the microscopic slide. A total of 3-μl drops of the resulting oocyst-agarose suspension were rapidly dispensed onto the prepared wells of the slide and distributed evenly using a pipette tip. For blocking, 20 μl of PBS-B was dispensed onto each well, and slides were incubated overnight at room temperature in a humidified chamber with slight agitation. For acetone permeabilization, slides were placed in a vertical microscopic slide holder filled with ice-cold acetone and incubated at −20°C for 30 minutes. The slides were then removed from the holder, and acetone was evaporated on air at room temperature. Then, either 7.5 μl of CLR binding buffer (50 mM HEPES, 5 mM MgCl_2_, and 5 mM CaCl_2_) and 2.5 μl of a 10-ng/μl CLR solution, 10 μl of a 1:25 dilution of 3G4 antibody, or 10 μl of a 2-μg/ml MPA-FITC solution were added to each well. The slides were incubated in a humidified chamber at room temperature for 2 h without agitation and then washed three times with 20 μl PBS per well before 10 μl of a 1:500 diluted secondary antibody in PBS-B was pipetted onto each well. Slides were again incubated for 2 h at room temperature in a humidified chamber. After washing as described above, 1.5 μl Fluoromount or Mowiol was pipetted into each well, which then was individually covered with a 12-mm round coverslip, and light pressure was applied and left for 10 minutes at room temperature for the mounting medium to harden.

### Preabsorption of MPA-FITC.

To test the specificity of suture staining, MPA-FITC was preabsorbed with either *N*-acetyl-galactosamine or *N*-acetylglucosamine. MPA-FITC was incubated with 100 mM sugars with slight agitation at RT overnight. Preabsorbed MPA-FITC was then incubated on blocked oocysts (see above) for 3 h. Then, oocysts were washed and processed for microscopy as described above.

### Uricase treatment.

Prior to the AEO-IFA procedure, oocysts or fluorescent beads, preincubated overnight with 0.5 mg/ml uric acid crystals, were incubated with 0.3-mg/ml uricase solution in a centrifuge tube for 90 min at 37°C. Oocysts and beads were then transferred to microscope slides and washed three times with PBS before the IFA procedure was continued as detailed above.

### Microscopy and imaging.

Counting of particles was performed with an Axioskop 2 microscope (Zeiss, Germany), equipped with an Axiocam for image acquisition. IFA for Fig. S1 was performed using a Zeiss Axioplan 2 epifluorescence microscope using a 63× oil immersion objective. Images were collected with an Axiocam digital camera using the Zeiss Axiovision 4.7 software. AEO-IFA slides were analyzed with an LSM-780 confocal laser scanning microscope (Zeiss) with Plan-Apochromat 63×/1.40 Oil DIC M27 and Plan-Apochromat 100×/1.40 Oil Ph3 M27 lenses. Images were obtained using Zeiss ZEN software. Excitation and emission wavelengths were adjusted to avoid carryover of fluorescence to other channels. Filters used included a UV filter for oocyst autofluorescence and MBS 488/543/633 for other fluorescent channels. For stacked images, the distance between the individual slices was set to 0.85 μm. Four to six slices from the middle of the z-stacks of the brightfield images were focus-stacked to obtain an image with a greater depth of field using the ImageJ Macro “Extended_Depth_of_Field.” (see Text S1 in the supplemental material). Fluorescent image z-stacks were projected onto a single plane with the max intensity setting of the ZProjection function of ImageJ 1.52a. Further processing of images for presentation was conducted using Adobe Photoshop CS6, Serif Affinity Photo 1.8.3, and Affinity Designer 1.8.3. For color-blind-friendly visualization, colors of the fluorescent channels were adjusted as follows: blue to cyan, red to magenta, and green to yellow (83).

10.1128/mSphere.01341-20.5 TEXT S1 ImageJ macro “Extended_Depth_of_Field.” Text S1, TXT file, 0.01 MB Copyright © 2021 Fabian et al. 2021 Fabian et al. https://creativecommons.org/licenses/by/4.0/ This content is distributed under the terms of the Creative Commons Attribution 4.0 International license.

10.1128/mSphere.01341-20.4 FIG S3 Montage of original images used to create Fig. 4. Stippled boxes indicate areas from which detailed oocyst representations were taken. The left column shows autofluorescence of AEO, the center column shows a signal of DyLight-650 labeled anti-human antibody in case of CLR binding, and the right column shows a merge of the two images. Brightness and contrast in merged images were adjusted equally for better visibility. For color-blind-friendly visualization, colors of the fluorescent channels were adjusted as follows: blue to cyan and red to magenta. Scale bar = 10 μm. Download FIG S3, TIF file, 1.6 MB. Copyright © 2021 Fabian et al. 2021 Fabian et al. https://creativecommons.org/licenses/by/4.0/ This content is distributed under the terms of the Creative Commons Attribution 4.0 International license.
