# Supplementary figures and images for: Expanding the Known Repertoire of C-Type Lectin Receptors Binding to Toxoplasma gondii Oocysts Using a Modified High-Resolution Immunofluorescence Assay
Source: mSphere. 2021 Mar 31;6(2):e01341-20. doi: 10.1128/mSphere.01341-20 (PMC8546727; doi:10.1128/mSphere.01341-20)

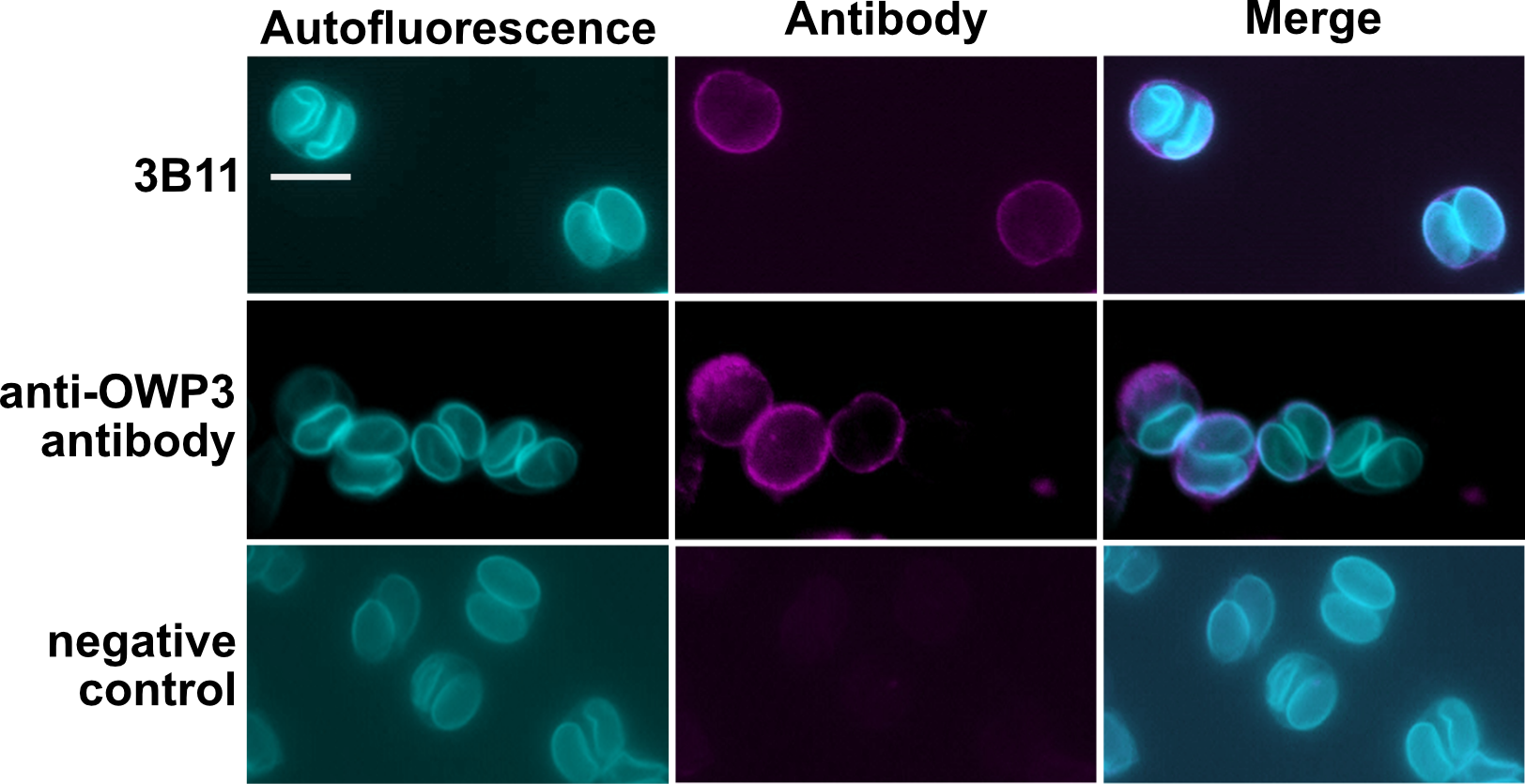

Supplement: FIG S1 [file msphere.01341-20-sf001.tif]

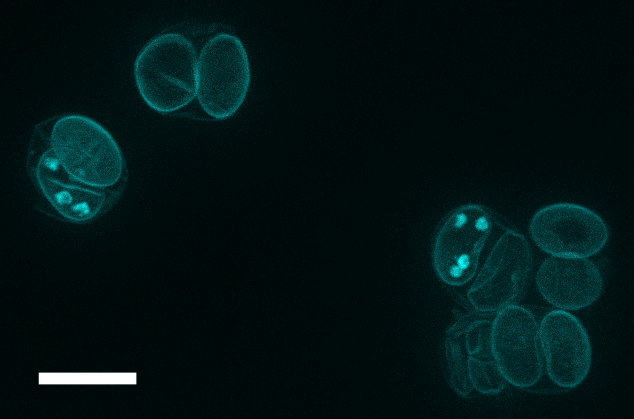

Supplement: FIG S2 [file msphere.01341-20-sf002.tif]

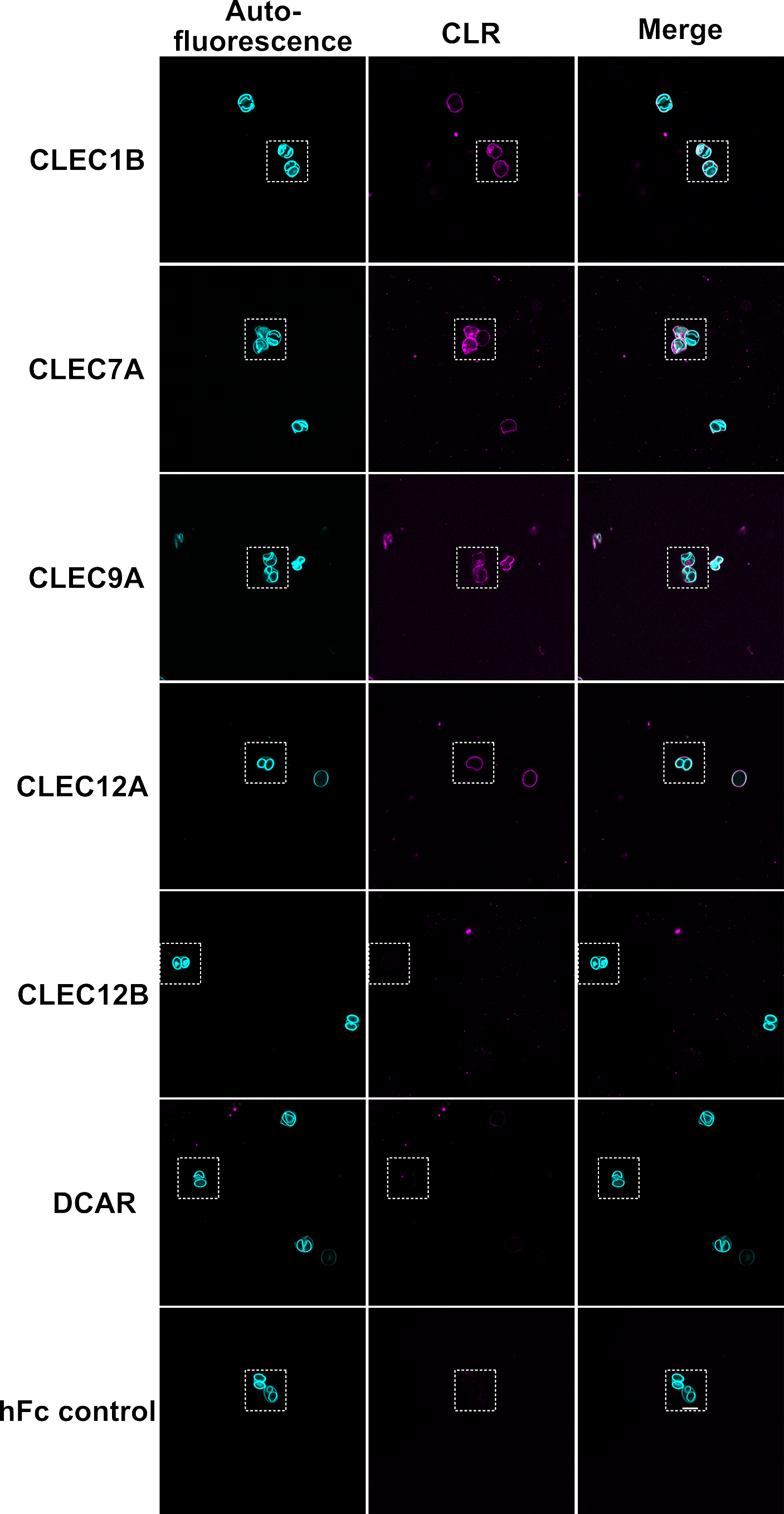

Supplement: FIG S3 [file msphere.01341-20-sf003.tif]
