# Supplementary material for: Expanding the Known Repertoire of C-Type Lectin Receptors Binding to Toxoplasma gondii Oocysts Using a Modified High-Resolution Immunofluorescence Assay
Source: mSphere. 2021 Mar 31;6(2):e01341-20. doi: 10.1128/mSphere.01341-20 (PMC8546727; doi:10.1128/mSphere.01341-20)
Supplement: TABLE S1 [file msphere.01341-20-st001.docx]

**Supplementary Table S1:** ELISA and immunofluorescence reactivity of anti-TgOWP3 antibodies.

| **antibody** | **OD** | **IF intensity^a^** |
| --- | --- | --- |
| mAb 3B11 | 0.95 | ++ |
| mAb 1G12**^b^** | 0.84 | (++) **^b^** |
| polyclonal anti-OWP3 | 1.90 | +++ |
| negative control | 0.12 | - |

^a^ See Fig. S1; **^b^** See Possenti A, Cherchi S, Bertuccini L, Pozio E, Dubey JP, Spano F. 2010. Molecular characterisation of a novel family of cysteine-rich proteins of Toxoplasma gondii and ultrastructural evidence of oocyst wall localisation. Int J Parasitol 40:1639-1649.
